# Problems related to levodopa‐carbidopa intestinal gel treatment in advanced Parkinson's disease

**DOI:** 10.1002/brb3.737

**Published:** 2017-06-05

**Authors:** Marianne Udd, Jukka Lyytinen, Johanna Eerola‐Rautio, Anu Kenttämies, Outi Lindström, Leena Kylänpää, Eero Pekkonen

**Affiliations:** ^1^ Department of Surgery Unit of Therapeutic Endoscopy Helsinki University Helsinki Finland; ^2^ Clinical Neurosciences, Neurology Helsinki University Helsinki Finland; ^3^ Department of Neurology Helsinki University Hospital and Helsinki University Helsinki Finland; ^4^ HUS Medical Imaging Center Helsinki University Helsinki Finland

**Keywords:** complication, duodopa, LCIG, mortality, Parkinson's disease, PEG‐J, weight loss

## Abstract

**Background:**

Continuous levodopa‐carbidopa intestinal gel (LCIG) diminishes daily “off” time and dyskinesia in patients with advanced Parkinson′s disease (PD). Complications are common with percutaneous endoscopic gastrostomy with a jejunal extension tube (PEG‐J).

**Aim of the Study:**

To report the clinical outcome of LCIG in patients with advanced PD in the years 2006–2014 at Helsinki University Hospital.

**Patients and Methods:**

Levodopa‐carbidopa intestinal gel treatment started following PEG‐J placement in patients with advanced PD after successful in‐hospital LCIG trial with a nasojejunal tube. Demographics, PEG‐J procedures, discontinuation of LCIG, complications and mortality were retrospectively analyzed.

**Results [mean (SD)]:**

Sixty patients with advanced PD [age 68(7) years; duration of PD: 11(4) years] had LCIG treatment for 26(23) months. The majority of patients with advanced PD were satisfied with the LCIG treatment. For 51 patients (85%), the pump was on for 16 hr a day, and for nine patients (15%) it was on for 24 hr a day. After 6 months, the levodopa‐equivalent daily dose (LEDD) had increased by 30% compared to pre‐LCIG LEDD. Sixty patients underwent a total of 156 PEG‐J procedures, and 48 patients (80%) had a total of 143 complications. Forty‐six patients (77%) had 119 PEG‐J or peristomal complications, and 22 patients (37%) had a total of 25 other complications. The most common complications were accidental removal of the J‐tube in 23 patients (38%) and ≥5% weight loss in 18 patients (30%). Fifteen patients discontinued the LCIG after 21 (21) months. At the end of the follow‐up period of 33(27) months, 38 patients were still on LCIG and nine (15%) had died.

**Conclusion:**

Most patients were satisfied with LCIG treatment. A few patients lost weight whereas the majority had complications with PEG‐J. When LCIG treatment is carried out, neurological and endoscopic units must be prepared for multiple endoscopic procedures.

## 1 INTRODUCTION

Patients with advanced Parkinson's disease (PD) suffer from daily motor fluctuations and dyskinesia. “On” time means periods of good motor control with no disturbing dyskinesia, while “off” time is periods of stiffness and poor mobility. Ideal medication reduces “off” time and minimizes dyskinesia. Combinations of levodopa with carbidopa or benserazide, dopamine agonists, MAO‐B inhibitors and COMT inhibitors are the standard treatment for PD (Horstink et al., 2006). In the advanced disease state, wearing off, on‐off phases, or dyskinesia lead to functional impairment (Ahlskog & Muenter, 2001). The short half‐life of oral levodopa and individual variation of gastric emptying in PD patients cause daily fluctuations in levodopa plasma concentration. Deep brain stimulation (DBS) (Deep Brain Stimulation for Parkinson's Disease Study, Group, 2001), apomorphine infusion (Trenkwalder et al., 2015) and continuous infusion of levodopa‐carbidopa intestinal gel (LCIG) (Nilsson, Nyholm, & Aquilonius, 2001) are device‐aided therapies that can diminish “off” ‐time and dyskinesia in advanced PD. DBS, LCIG and an apomorphine pump are considered only if a combinations of the aforementioned oral drugs is not sufficient. In Finland DBS has been available since 1995, and LCIG since 2006 with 100% reimbursement. Apomorphine pump has been available in Finland since April 2017.

Levodopa‐carbidopa intestinal gel with a levodopa concentration of 20 mg/ml is administered via a portable pump that is connected to the PEG‐J, a percutaneous endoscopic gastrostomy (PEG) with a thinner inner J‐tube placed in the proximal jejunum. LCIG treatment ensures continuous dopaminergic stimulation and it significantly reduces daily motor fluctuations and dyskinesia compared to oral levodopa (Olanow et al., 2014). However, complications with the tube or the pump are common, presenting in 40%–96% of patients (Devos, 2009; Fernandez et al., 2013, 2015; Nyholm et al., 2008; Pickut, van der Linden, Dethy, Van De Maele, & de Beyl, 2014).

The aim of the present study was to analyze the outcome of long‐term LCIG treatment in advanced PD, paying special attention to complications and discontinuation of the treatment in the clinical setting.

## 2 PATIENTS AND METHODS

Altogether, 60 patients with advanced PD received LCIG therapy at Helsinki University Hospital between 2006 and 2014. A neurologist selected candidates for LCIG treatment i.e., patients who reported substantial daily motor fluctuations and dyskinesia that could not be sufficiently controlled with oral PD medication. Severe dementia, ongoing psychosis, and unresponsiveness to levodopa were exclusion criteria. The total daily oral doses of levodopa, amantadine, dopamine agonists and MAO‐B‐ and COMT‐inhibitors were converted to a morning dose and continuous daily infusion of LCIG. To assess the response to LCIG, the patients were admitted to neurological ward and had a nasojejunal tube (Flocare Bengmark^®^ 10Fr, Nutricia, Netherlands) for 4–6 days of testing. A radiologist positioned the tube near the ligamentum of Treitz under fluoroscopic control. LCIG was administered with a pump via the nasojejunal tube, and the response to LCIG was assessed with diaries***.*** If this testing phase was successful, the patient had a PEG‐J.

### 2.1 PEG‐J procedure

A PEG‐J tube (Freka^®^ 15 Fr or 20 Fr PEG ‐tube and Freka^®^ 9 Fr intestinal Tube, Fresenius Kabi, Cheshire, UK) was inserted under endoscopic and fluoroscopic control by gastroenterologic surgeons in the endoscopic unit using the pull‐through method (Gauderer, Ponsky, & Izant, 1980). Antibiotic prophylaxis (1.5 g cefuroxime, Zinacef^®^, GlaxoSmithKline, Espoo, Finland) and local anesthesia with lidocaine (10 mg/ml Lidocain^®^, Orion, Espoo, Finland) were administered, and conscious sedation was provided by an anesthesiologist. After placement of the PEG tube, the inner J tube was moved near the ligamentum of Treitz with rat‐tooth forceps. If this failed, a guidewire (Jagwire, Boston Scientific, Alajuela, Costa Rica) and a triple lumen balloon were passed to the duodenum, the balloon was retrieved and the J tube inserted over the guidewire. The position of the inner tube was controlled under fluoroscopy. The PEG‐J was connected to a portable infusion pump (CADD legacy 1400 Duodopa pump, Smits Medical ASD, St Paul, MN, USA).

### 2.2 LCIG infusion in practice

The LCIG (20 mg/ml of levodopa and 5 mg/ml carbidopa; Duodopa^®^, Abbvie) dose optimization was carried out in the neurologic ward. All oral PD medication was usually stopped, with the exception of high‐dose dopamine agonists with positive clinical response. The morning bolus was followed by a continuous infusion. Additional doses were administered by the patient when they felt they were entering an “off” phase. Usually, infusion lasted 16 hr, supplemented with a sustained‐release oral dose of levodopa for the night. If necessary, night infusion was applied, with the infusion rate being about 40%–60% less than infusion during the day. Control phone calls were planned for 2–4 weeks after and control visits for 6 months after initiation of LCIG for clinical evaluation in the outpatient clinic. The patients were advised to gradually increase the daily infusion if several extra doses had to be taken daily. In cases of inner tube problems, the patients were advised to administer LCIG via PEG to the gastric space or to restart oral medication. If an infusion problem occurred, a scheduled admission to the neurological ward was arranged, with a subsequent endoscopic procedure to correct the tube problem.

### 2.3 Data collection at baseline and during the follow‐up

The following data were collected: duration of PD, age at onset of PD, preceding PD medication, ASA (Physical Status Classification of the American Society of Anesthesiologists) class, body mass index (BMI), concomitant diseases, details of the PEG‐J procedure, living conditions (alone, with a spouse, in institutional care), a mini‐mental state examination (with MMSE ≥25 indicating normal cognition), a Hoehn and Yahr scale assessment, neurosurgical contraindications (coagulopathy, cognitive impairment). Also daily hours on LCIG, LCIG doses after 6 months, and any additional oral medication data were collected. At the end of the follow‐up in November 2015, information about weight loss (weight change as a percentage during follow‐up), living conditions and discontinuation of the LCIG treatment was gathered. The number of contacts with the stoma nurse, data of PEG‐J related (tube occlusion, accidental removal of inner tube, dislocation of the inner tube backwards into the stomach, tube breakage), peristomal (stoma leakage, granulation tissue around stoma, skin excoriation, abscess or infection, PEG tube hat buried in gastric wall, i.e., buried bumper syndrome (BBS)), or other (≥5% weight loss, gastric ulceration caused by inner tube, neurological) complications and mortality were collected from the patient files for the study. Underweight was defined as a BMI of <18.5 m^2^/kg, normal weight as a BMI 18.5–25 m^2^/kg, and overweight as a BMI of >25 m^2^/kg.

### 2.4 Informed consent statement

The study was approved by the hospital ethics committee. Data extraction occurred retrospectively from hospital medical records. According to Finnish law, retrospective research using hospital medical files does not require informed consent from the study subjects.

### 2.5 Statistics

The results are reported as means and standard deviation (*SD*). The significance of differences in categorical data was determined using Fisher's exact test. The Mann–Whitney *U* test was used to discover the differences in continuous variables. A level of *p *<* *.05 was regarded as statistically significant, and two tailed tests were used. Statistical calculations were generated using IBM SPSS Statistics 21 (International Business Machines Corporation, Endicott, NY, USA).

## 3 RESULTS

Mean age at onset of PD was 56 (8) years, and the duration of PD was 11 (4) years before LCIG treatment. There were 32 men (53%) and 28 women (47%) with a mean age of 68 (7) years, and 27 participants (45%) were 70 or older. Baseline characteristics are presented in Table 1. The mean Hoehn and Yahr score in the “on” phase was 2.7 (0.7). At the onset of LCIG treatment, all the patients had been on levodopa only, or a combination of amantadine, a dopamine agonist, and MAO‐B‐ or COMT‐ inhibitors, with a mean levodopa‐equivalent daily dose (LEDD) of 1,266 (441) mg. Ten patients (17%) were taking only levodopa, 18 (31%) were taking two drugs and 30 (52%) had three or more different drugs daily. An MMSE test was performed on 48 patients prior to LCIG treatment. The mean MMSE score was 26 (3). Altogether, 29 patients refused or had contraindications for DBS treatment. The details of the PEG‐J procedure are presented in Table 2.

**Table 1:** Baseline characteristics of PD patients on LCIG treatment

Demographics	*n* = 60
ASA I	0
ASA II	3 (5%)
ASA III	51 (85%)
ASA IV	6 (10%)
Coronary/‐heart disease	17 (28%)
Diabetes	8 (13%)
Psychiatric diagnosis	11 (18%)
MMSE ≤24	11 (23%)
Hoehn and Yahr score on phase ≥4	6 (10%)
Lives with a spouse	44 (73%)
Lives alone	12 (20%)
In sheltered housing with assistance	4 (7%)
Walker as mobility aid	15 (25%)
Wheelchair or crutches as mobility aid	6 (11%)

PD, Parkinson's disease; LCIG, levodopa‐carbidopa intestinal gel; ASA, data of physical status classification of the American Society of Anesthesiologists ASA class; MMSE, mini‐mental state examination.

**Table 2:** PEG‐J placement procedure in 60 patients

Time from nasojejunal test tube placement to PEG‐J placement; days; mean (*SD*)	6.5 (2.7)
Total hospital stay, days; mean (*SD*)	11 (4)
Hospital stay after PEG‐J placement, days	3.9 (3.9)
Length of the procedure, min; mean (*SD*)	31 (16)
Antibiotic prophylaxis	48 (87%)
PEG‐J: Fresenius Freka^®^ 15 Fr	51 (85%)
PEG‐J: Boston^®^ 20 Fr	9 (15%)
Inner tube in the descending or transverse duodenum	6 (10%)
Inner tube in the ligament of Treitz	54 (90%)

PEG‐J, percutaneous endoscopic gastrostomy with jejunal tube; *SD*, standard deviation.

### 3.1 LCIG dose

After discharge, 51 patients (85%) had daily LCIG infusion, and nine cases (15%) were 24 hr a day. The mean daily LCIG dose was 1,651 (595) mg. At 6 months, 42 patients (78%) had daily infusion and 12 (22%) had 24‐hr infusion. In most cases (44), the LEDD had increased by 505 (304) mg compared with the baseline. In nine patients, the LEDD had decreased by 255 (126) mg. Doses of LCIG at baseline and 6 months were: morning bolus 191 (67) mg vs. 170 (71) mg, continuous infusion 73 (27) mg/hr vs. 81 (29) mg/hr (6 a.m.–10 p.m.) and 57 (26) mg/hr vs. 72 (43) mg/hr (10 p.m.–6 a.m.), correspondingly.

At the end of the follow‐up period of 33 (24) months, 38 patients (63%) were on LCIG, 7 (12%) had been on LCIG until death, 13 (22%) had discontinued LCIG and were alive, and two patients (3%) discontinued LCIG and died later.

Thirty‐two patients were on LCIG for more than 2 years, and 12 patients for more than 4 years. Fifty‐three patients (90%) felt that LCIG treatment still substantially alleviated motor symptoms, when questioned at 6 months and beyond.

### 3.2 Changes in weight, living conditions and need for walking devices during LCIG treatment

At baseline, the mean BMI of the patients was 24.7 (4.2) kg/m^2^: only three patients (5%) were underweight, 31 (57%) were normal weight and 26 (43%) were overweight. At the end of the follow‐up, eight patients (13%) were underweight, 31 (52%) were normal weight and 21 (35%) were overweight. The weight change as a percentage was −3.3% (10.7%) in 25.7 (23.1) months. Eighteen patients (30%) had weight loss of ≥5%, and in 12 patients (20%), weight loss was ≥10%. One patient had fatal weight loss despite discontinuation of LCIG. Body CT did not show any malignancy. The autopsy failed to reveal any clear cause for his deterioration. Two patients had peripheral neuropathy prior to LCIG, probably due to diabetes and spinal stenosis.

At the end of the follow‐up, 38 patients (66%) were still living at home with their spouse or alone, 17 (29%) were in institutional care, (mainly sheltered housing with 24‐hr assistance), and three patients had intervals at home and in institutions. Nine patients originally living with their spouse (21%) and four originally living alone (33%) had moved to sheltered housing. The need to use a walker increased by 40%, (*n* = 21, 37%) and a wheelchair by 50% (*n* = 6; 11%), and two patients became bedridden.

### 3.3 Discontinuation

Cognitive decline or dementia at the baseline or appearance of these symptoms during follow up were the most common causes of infusion withdrawal, occurring in seven patients after 27 (25) months of LCIG. In two bedridden patients living in institutions, LCIG was stopped after 38 (12) months. One patient was suffering from stoma problems and discontinued the treatment at 19 months. Two patients (3.3%) stopped LCIG after 3 months and 5 months because of inefficacy. One patient subjectively felt dizziness during LCIG treatment and decided to discontinue the LCIG infusion after a month, although clinical examination showed no signs of either postural instability or orthostatic hypotension. Altogether, after a mean of 21 (21) months, LCIG was discontinued in 15 patients, and in 11 cases the reason was recurrent removal of the inner tube by the patient.

### 3.4 Complications

Sixty patients underwent a total of 156 endoscopic procedures. An additional 96 endoscopic procedures following 60 PEG‐J placements are presented in Table 3, and complications related to LCIG treatment are shown in Table 4. There were 48 patients (80%) with a total of 143 complications, and only 12 patients with no complications. Altogether, 46 patients (77%) had a total of 119 PEG‐J or peristomal complications, and 22 (37%) had a total of 25 other complications. On average, patients had 2.4 (2.1) complications. In 30 days after PEG‐J, 11 patients (18%) had complication (six peristomal infections, one granulation, one gastric hematoma, one nonspecific infection, one knot and occlusion, and one disorientation)**.** Thirty‐one patients visited a stoma nurse a total of 121 times. Accidental removal of the inner tube occurred significantly more often in patients with cognitive decline (MMSE <24), than in those without it: 8 (73%) vs. 12 (35%); *p* = .034. There were no peritonitis cases during the follow‐up.

**Table 3:** Additional procedures after PEG‐J placement in the endoscopy unit (*n* = 96)

Hospital stay, days; mean(*SD*)	2.3 (3.7)
Length of the procedure, min; mean (*SD*)	19 (11)
Indications for the procedure	
Accidental removal of inner tube	37 (38%)
Tube occlusion	27 (29%)
Tube break	14 (15%)
Stoma leak	4 (4%)
Dislocation of the inner tube backwards into the stomach	2 (2%)
Thicker PEG‐J for nutrition	4 (4%)
Discontinuation of the treatment	8 (8%)
Procedures: *n* = 96	
Inner tube placement or exchange	45 (47%)
PEG‐J tube exchange	25 (26%)
Testing the tube, checking with fluoroscopy or gastroscopy, exchanging the caps	13 (14%)
Removal of the PEG‐J system^a^	13 (14%)

PEG‐J, percutaneous endoscopic gastrostomy‐jejunal tube; *SD*, standard deviation.

a Of 15 patients discontinuing LCIG, two patients used the PEG for nutrition and it was not removed.

**Table 4:** Complications in 60 patients on LCIG

Complication	*n* = 60
Peristomal complications:	
Buried PEG bumper	1 (1%)
Skin problems, leaking stoma	12 (20%)
Nonspecific infection	4 (7%)
Skin infection, abscess	5 (8%)
Granulation tissue	21 (35%)
Tube complications:	
Tube occlusion	13 (22%)
Accidental removal of inner tube	23 (38%)
Dislocation of the inner tube backwards into the stomach	5 (8%)
Tube break	11 (18%)
Other complications:	
Weight loss ≥5%	18 (30%)
Neurologic symptoms	3 (5%)
Pump issue	3 (5%)
Peritonitis	0

LCIG, levodopa‐carbidopa intestinal gel.

### 3.5 Mortality

According to death certificates provided by Statistics Finland, there were no PEG‐J related complications or deaths. Altogether, nine patients died, seven of whom were on LCIG until death. Two patients died 3.7 and 21.5 months after discontinuing LCIG, respectively. The time from the start of LCIG to death was 26.6 (14) months. The causes of death were defined by clinical examination in four patients and by clinical autopsy in five patients. The immediate causes of death were pneumonia (*n* = 4), advanced PD (*n* = 2), coronary heart disease or insufficiency (*n* = 2) and pulmonary embolism (*n* = 1). The underlying causes of death were PD (*n* = 7) and coronary heart disease (*n* = 2).

Body mass index at onset was significantly higher in those alive at the end of the follow‐up compared to those who died: 25.1(4.4) vs. 22.2(3.4); *p* = .043. Similarly, BMI at the latest follow‐up visit was significantly higher in those alive at the end of the follow‐up than in those who died during the follow‐up, at 24.3 (4.0) vs. 18.9 (3.1); *p* = .001. Four underweight patients (44%) died, compared to five patients (8%) with normal weight or overweight; *p* = .013.

## 4 DISCUSSION

Levodopa‐carbidopa intestinal gel treatment has proved to be effective in reducing levodopa‐related dyskinesia and diminishing off time compared to oral medication, leading to improvement in the quality of life (Antonini, Yegin, Preda, Bergmann, & Poewe, 2015; Lopiano et al., 2016; Olanow et al., 2014; Wirdefeldt, Odin, & Nyholm, 2016). We present data on LCIG therapy, focusing on complications. The majority of our patients were satisfied with the infusion during follow‐up. There were, however, numerous tube and stoma complications related to LCIG, as reported previously (Fernandez et al., 2015; Lang et al., 2016; Nilsson et al., 2001).

In most of our patients, LEDD was increased at 6 months compared to baseline. The LEDD increase may be caused by disease progression. We had 12 patients (20%) with their LCIG pump running for 24 hr. That is slightly more than previously reported by Devos et al. (10% LCIG for 24 hr) (Devos, 2009). One study showed a quite stable LEDD on LCIG for 12 months (Antonini et al., 2015).

In some studies, dementia was an exclusion criterion, and only patients with an MMSE of 28–29 were included (Epstein et al., 2016; Fernandez et al., 2015). In a French multicentre study (Devos, 2009), 50% of the patients on LCIG had cognitive disorders suggestive of PD dementia. We found LCIG suitable for patients with mild dementia living with a motivated spouse. However, there was more accidental tube removal in patients with cognitive decline. Hence, patients with dementia living alone appear not to be suitable candidates for LCIG. Despite the LCIG treatment, 29% of our patients were in institutional care, mainly sheltered housing with 24‐hr assistance at the end of the follow‐up.

**Figure 1:**
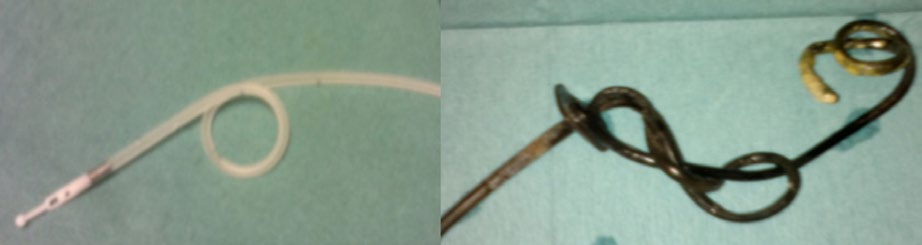
Naive and knotted jejunal tube

Several technical problems and complications increase the annual admission rate and contact with the hospital (Nyholm et al., 2008). Complications with the PEG‐J tube (Devos, 2009; Fernandez et al., 2013; Nyholm et al., 2008) are similar to complications related to PEG for feeding purposes (Schapiro & Edmundowicz, 1996; Udd et al., 2015). The risk of peritonitis has varied between zero and 4% (Devos, 2009; Epstein et al., 2016; Lang et al., 2016; Palhagen et al., 2016), and other serious complications like colonic perforations, gastropleural fistula (Klostermann et al., 2012) and liver injury (Pickut et al., 2014), have also been described. In our material, we did not have any peritonitis, nor any need for emergency abdominal surgery due to LCIG complications. A peristomal infection risk of 8% is comparable with the literature: 10%–20% (Devos, 2009; Epstein et al., 2016; Fernandez et al., 2015; Olanow et al., 2014). Inner tube complications were mostly accidental removal, kinking or dislocation of the tube occurring during the LCIG treatment in physically active patients, sometimes suffering from disorientation (Devos, 2009; Fernandez et al., 2013; Nyholm et al., 2008; Pickut et al., 2014). We did not find any cases of inner tube‐induced duodenal decubitus ulcer (Martino et al., 2016), or bezoar sometimes present in these patients. Our results showed that 13 patients had a total of 27 tube occlusions, and eight of them (61%) had altogether ten knots in the inner tube. Previously, only three case reports of five patients with knotting of the inner tube had been published (del‐Hoyo‐Francisco et al., 2015; Krones, Zollner, & Petritsch, 2012; Nyholm et al., 2008). The Freka^®^ intestinal tube has an angled, C‐shaped tip, and this may predispose the tube to knots (Figure 1). The present results showing that removal of the inner tube occurs more often in patients with dementia are supported by previous findings (Devos, 2009). Logically, among patients with no dementia, the rate is lower (Epstein et al., 2016; Fernandez et al., 2015).

The triangular external fixation plate of the Freka^®^ PEG‐J tube is suboptimal, because it glides along the PEG tube, allowing the PEG to move back and forth. This movement may even predispose the patient to peritonitis in the first days when the stoma is maturing (Epstein et al., 2016). The skin around the peristomal area may become irritated by leaking gastric fluids. Granulation formation needs referral to a stoma nurse. Granulation tissue was more common in our patients (35%) compared to other series (20%–22%) (Epstein et al., 2016; Lang et al., 2016).

Weight loss is an adverse event increasing the risk of death, and is partly associated with the natural course of PD. One third of our patients had ≥5% weight loss during the follow‐up with one fatal weight loss despite discontinuation of LCIG. Decreased weight has been observed in 5%–10% of LCIG patients (Antonini et al., 2015; Merola et al., 2016). The mechanism for weight loss in LCIG remains ambiguous. Monitoring weight during LCIG treatment is essential to avoid serious weight loss.

Eighty percent of the patients had complications leading to multiple endoscopic procedures, but still 90% were satisfied with the treatment. Some of the complications may therefore be related to the suboptimal devices and tubes. A new T‐port was found to be well tolerated, and it had a low number of tube problems, but proper cleaning and local treatment of the stoma site were necessary (van Laar, Nyholm, & Nyman, 2016). The use of a T‐port is not widely spread, though.

Currently, there are no guidelines for withdrawal of LCIG. If the patient is bedridden, disoriented and already needs maximal care, LCIG probably may not give any significant health benefit. Several removals of the inner tube by a patient during the treatment suggest that the mental condition of that patient has significantly deteriorated. In these cases, discontinuation of LCIG should be individually considered. Further, several replacements of the inner tube increase the burden of already limited hospital resources. Continuous weight loss can even be a fatal complication, and therefore needs monitoring. Close co‐operation between neurological and endoscopic units is required, and units must be prepared for common and recurrent PEG‐J problems when LCIG treatment for advanced PD is carried out.

## CONFLICTS OF INTEREST

Marianne Udd received a one‐month researcher's salary from the Helsinki University Hospital Research Fund. The authors have no conflicts of interest to disclose.
